# Supplementary material for: Effects of Aging on Hair Color, Melanosomes, and Melanin Composition in Japanese Males and Their Sex Differences
Source: Int J Mol Sci. 2022 Nov 21;23(22):14459. doi: 10.3390/ijms232214459 (PMC9693441; doi:10.3390/ijms232214459)
Supplement: Supplementary file 1 [file ijms-23-14459-s001.zip › ijms-2027964-supplementary.pdf]

Supporting Information

Effects of Aging on Hair Color, Melanosome, and Melanin Composition in Japanese Males and Their Sex Differences

Takashi Itou [1], Shosuke Ito [2] and Kazumasa Wakamatsu [2]

[1] Kao Corporation, R&D - Hair Care Products Research, 2-1-3, Bunka, Sumida-ku, Tokyo 131-8501, Japan

[2] Institute for Melanin Chemistry, Fujita Health University, 1-98, Dengakugakubo, Kutsukake-cho, Toyoake, 470-1192, Japan

Table S1. The hair samples and the determined values of hair color and major and minor axis of the isolated melanosomes.

Sample name	Age (year)	Hair tress			Melanosome		
		CIELAB coordinates			[1]n	Mean ± SD (μm)	
		*L**	*a**	*b**		Major axis	Minor axis
DM-1	4	15.9	4.0	5.5	235	0.88±0.14	0.32±0.05
DM-21	4	15.7	2.8	4.0	257	0.89±0.15	0.34±0.06
DM-22	5	17.5	2.9	3.9	253	0.91±0.17	0.33±0.06
HD05-62	6	15.0	3.0	3.6	315	0.92±0.20	0.37±0.08
DM-2	11	14.7	1.4	2.4	272	0.88±0.16	0.35±0.07
HS019	12	16.5	2.8	3.6	419	0.91±0.17	0.39±0.09
DM-23	12	18.0	2.3	3.1	219	0.88±0.18	0.34±0.07
DM-3	16	15.4	1.5	2.1	213	0.82±0.18	0.39±0.10
DM-24	19	13.3	1.5	2.5	233	0.83±0.16	0.33±0.06
DM-4	20	15.8	1.6	2.2	249	0.75±0.14	0.33±0.07
DM-25	21	14.9	2.4	3.1	229	0.92±0.18	0.36±0.08
HD106	28	17.0	2.5	3.4	268	0.88±0.18	0.37±0.08
HD178	28	14.7	1.8	2.4	220	0.96±0.18	0.39±0.08
DI	28	14.1	2.2	2.8	242	0.97±0.19	0.36±0.07
GK	28	13.9	2.1	2.8	307	0.97±0.22	0.41±0.09
SW	30	16.7	3.1	4.1	234	0.89±0.18	0.35±0.07
DM-26	30	16.1	0.8	0.9	257	0.93±0.16	0.40±0.08
BB-1	31	16.0	2.3	2.8	325	0.91±0.16	0.35±0.07
DM-6	33	15.6	3.3	4.2	239	0.87±0.16	0.39±0.09
DM-7	33	16.8	2.0	2.5	234	0.81±0.16	0.35±0.08
DM-8	34	16.2	2.7	3.5	293	0.89±0.15	0.35±0.07
DM-27	34	14.3	1.5	2.1	312	0.94±0.21	0.36±0.07
HD21	36	16.4	3.2	4.2	314	0.80±0.17	0.33±0.07
DM-28	36	14.6	2.0	2.7	208	0.90±0.17	0.39±0.09
SK001	39	15.7	2.3	2.8	203	0.88±0.16	0.36±0.08
BB-2	39	12.3	1.9	2.6	430	0.90±0.18	0.38±0.09
DM-29	41	15.3	1.8	2.5	229	0.89±0.16	0.38±0.08
MT-1	42	-	-	-	265	0.85±0.15	0.34±0.07
HD149	43	15.4	1.4	1.8	220	0.86±0.16	0.37±0.09
DM-30	44	15.5	1.2	1.6	256	0.94±0.17	0.37±0.07
DM-10	50	13.8	0.8	1.4	233	0.93±0.15	0.39±0.09
DM-31	50	14.8	2.5	3.5	202	0.99±0.20	0.43±0.12
DM-32	50	15.9	1.5	1.8	286	0.94±0.18	0.43±0.10
MT-2	50	13.7	1.9	3.1	307	0.97±0.22	0.41±0.09
DM-33	51	14.6	1.4	2.3	210	0.94±0.20	0.42±0.10
DM-11	52	15.5	1.1	1.8	243	0.82±0.14	0.38±0.08
DM-13	52	15.4	1.5	1.9	276	0.89±0.16	0.42±0.09
DM-14	53	15.7	2.1	3.0	213	0.93±0.18	0.41±0.09
MT-3	55	13.1	1.3	2.4	208	0.89±0.18	0.34±0.06
DM-36	58	14.1	1.6	2.2	208	0.94±0.17	0.37±0.09
TI	60	-	-	-	269	0.98±0.17	0.40±0.09
DM-17	60	14.1	1.1	1.7	203	0.89±0.17	0.40±0.09
DM-18	68	14.6	0.5	0.9	241	0.93±0.19	0.42±0.11
HD20	72	13.4	1.7	2.4	271	1.01±0.18	0.45±0.11

[1]n, the number of the isolated melanosomes used in the size measurements.

Table S2. Levels of various melanin markers in the hair samples.

Sample name	Age (year)	Soluene-350 Solubilization		H_2O_2 oxidation (ng/mg)				H_2O_2 oxidation after HCl hydrolysis (ng/mg)				4-AHP (ng/mg)	TM (μg/mg)	Pheo-melanin (mol%)	DHI (mol%)
		A500 (mg^{-1})	A650/A500 ratio	PTCA level	PDCA level	TTCA level	PTeCA level	PTCA level	PDCA level	TDCA level	PTeCA level				
DM-1	4	0.211	0.294	222	24.5	40.8	75.8	113	16.5	44.5	78.8	2.7	21.3	13.0	70.6
DM-21	4	0.156	0.308	196	22.9	40.4	51.9	74.9	12.0	39.3	51.0	3.1	15.8	15.7	72.3
DM-22	5	0.141	0.326	147	18.8	36.3	42.6	63.8	10.1	36.4	45.4	3.6	14.2	17.3	75.0
HD05-62	6	0.177	0.294	200	16.4	47.6	67.9	92.8	11.2	34.5	75.7	1.6	17.9	14.8	63.2
DM-2	11	0.189	0.317	227	24.6	39.4	57.8	103	17.9	41.0	70.1	2.7	19.1	11.0	70.1
HS019	12	0.159	0.302	202	16.4	47.3	64.3	94.8	11.2	38.1	87.8	3.0	16.1	16.4	62.9
DM-23	12	0.169	0.308	168	15.6	32.9	32.7	77.0	12.5	35.9	50.9	2.4	17.1	13.8	66.0
DM-3	16	0.214	0.299	246	29.6	48.8	90.6	115	17.0	49.1	89.4	3.6	21.6	13.9	73.2
DM-24	19	0.189	0.307	224	25.0	52.1	57.2	93.8	15.6	48.4	65.0	5.1	19.1	14.9	70.9
DM-4	20	0.178	0.292	222	23.2	54.4	58.9	85.8	19.7	40.0	55.4	20.9	18.0	9.8	69.1
DM-25	21	0.155	0.284	208	19.4	34.6	60.2	86.8	13.6	33.1	56.4	3.3	15.7	11.7	66.1
HD106	28	0.192	0.302	198	18.4	48.7	67.2	82.7	10.7	39.8	72.2	3.1	19.4	17.9	66.0
HD178	28	0.166	0.295	194	18.4	52.0	67.9	85.4	10.2	39.6	69.3	2.6	16.8	18.7	66.5
DI	28	0.188	0.287	207	18.4	56.2	82.4	97.3	11.2	37.5	82.9	4.1	19.0	16.1	65.0
GK	28	0.196	0.281	235	33.8	48.8	83.4	111	16.1	41.4	80.3	3.5	19.8	12.4	75.7
SW	30	0.163	0.307	191	16.4	40.4	53.3	84.0	9.2	35.5	65.0	2.3	16.5	18.6	64.2
DM-26	30	0.264	0.303	306	40.0	55.6	91.0	124	21.3	54.8	84.1	4.1	26.7	12.4	75.1
BB-1	31	0.236	0.314	328	24.5	67.0	103	121	13.3	42.0	89.9	3.4	23.8	15.2	61.2
DM-6	33	0.195	0.303	220	24.2	41.5	81.1	96.1	14.2	39.6	72.0	2.9	19.7	13.4	70.5
DM-7	33	0.160	0.300	191	23.9	45.6	61.6	74.5	18.8	37.4	48.9	5.0	16.2	9.6	74.5
DM-8	34	0.190	0.311	250	23.1	49.9	71.4	114	16.3	47.9	89.7	3.5	19.2	14.1	65.9
DM-27	34	0.241	0.282	238	31.6	55.9	74.1	98.0	19.7	50.3	71.0	3.3	24.3	12.3	75.2
HD21	36	0.119	0.319	140	12.3	64.7	37.7	61.3	8.7	27.4	48.4	3.0	12.0	15.1	64.7
DM-28	36	0.193	0.301	230	27.6	58.5	62.1	101	18.0	50.7	75.8	7.1	19.5	13.5	73.2
SK001	39	0.212	0.321	241	24.5	68.0	73.5	104	13.3	33.9	90.9	2.6	21.4	12.3	68.3
BB-2	39	0.237	0.304	326	24.5	73.2	104	137	16.4	50.5	108	2.8	23.9	14.8	61.4
DM-29	41	0.219	0.315	245	30.2	47.5	72.2	104	16.6	54.2	88.4	3.5	22.1	15.7	74.0
MT-1	42	0.188	0.293	231	16.4	58.8	85.3	100	11.3	38.2	72.2	4.5	19.0	16.3	60.3
HD149	43	0.176	0.318	214	20.4	70.4	73.5	91.5	11.2	43.2	78.8	3.9	17.8	18.5	66.7
DM-30	44	0.222	0.297	276	31.6	54.2	91.0	117	19.3	54.4	96.7	4.1	22.4	13.6	71.7
DM-10	50	0.298	0.326	293	33.7	55.6	75.8	117	21.9	56.6	92.5	4.2	30.1	12.4	71.8
DM-31	50	0.208	0.298	226	24.5	63.6	67.3	109	17.1	53.5	86.5	3.9	21.0	15.0	70.1
DM-32	50	0.226	0.310	209	27.2	42.0	67.3	89.7	18.1	41.3	65.2	2.7	22.8	11.0	75.1
MT-2	50	0.196	0.311	241	23.1	43.9	79.2	92.1	14.4	40.3	61.4	4.0	19.8	13.5	66.8
DM-33	51	0.219	0.311	225	27.7	49.9	59.3	91.7	18.2	46.9	67.2	4.4	22.1	12.4	74.0
DM-11	52	0.229	0.314	250	27.0	47.6	67.6	96.4	16.9	43.9	71.1	4.6	23.1	12.5	70.0
DM-13	52	0.216	0.301	232	29.6	54.2	79.4	98.1	15.0	46.5	72.0	4.3	21.8	14.9	75.0
DM-14	53	0.178	0.320	212	24.4	44.9	52.8	81.5	15.1	36.1	53.9	10.8	18.0	11.5	71.9
MT-3	55	0.208	0.322	244	28.9	44.2	65.4	91.9	15.5	41.4	63.6	3.8	21.0	12.8	72.7
DM-36	58	0.258	0.310	264	30.1	62.1	60.2	111	20.0	52.1	82.6	4.9	26.1	12.5	71.6
TI	60	0.286	0.311	340	36.8	87.5	97.2	141	19.4	67.8	117	3.2	28.9	16.8	70.1
DM-17	60	0.259	0.317	266	33.6	58.5	76.4	110	19.3	55.6	81.5	4.6	26.2	13.9	74.8
DM-18	68	0.309	0.324	327	41.6	55.4	81.7	128	24.1	58.6	93.4	4.8	31.2	11.7	75.0
HD20	72	0.257	0.311	280	24.5	75.1	110	130	17.9	60.1	110	3.8	26.0	16.1	64.6

PTCA: pyrrole-2,3,5-tricarboxylic acid, PDCA: pyrrole-2,3-dicarboxylic acid, PTeCA: pyrrole-2,3,4,5-tetracarboxylic acid, TTCA: thiazole-2,4,5-tricarboxylic acid, TDCA: thiazole-4,5-dicarboxylic acid, 4-AHP: 4-amino-3-hydroxyphenylalanine

Table S3. Results of the regression analyses between hair color parameters and various values for all (male+female) subjects.

Parameter	*L**		*a**		*b**	
	R^2	*p*-value	R^2	*p*-value	R^2	*p*-value
PTCA level (ng/mg)	0.43	2.0×10^{-9}	0.48	8.4×10^{-11}	0.48	7.2×10^{-11}
PDCA level (ng/mg)	0.36	9.7×10^{-8}	0.61	4.8×10^{-15}	0.59	4.0×10^{-14}
PTeCA level (ng/mg)	0.25	1.8×10^{-5}	0.14	0.0015	0.18	0.00037
TTCA level (ng/mg)	0.19	0.00027	0.18	0.00031	0.21	7.9×10^{-5}
4-AHP level (ng/mg)	0.0012	0.78	0.0045	0.59	0.0091	0.44
TM (μg/mg)	0.40	8.3×10^{-9}	0.52	4.6×10^{-12}	0.50	2.0×10^{-11}
DHI (mol%)	0.037	0.12	0.22	5.9×10^{-5}	0.20	0.00018
Pheomelanin (mol%)	0.13	0.0031	0.19	0.00020	0.17	0.00046

Table S4. Results of the multiple regression analyses among hair color parameters, TM, DHI mol%, and pheomelanin mol% for all (male+female) subjects.

Hair color parameter	Result		Intercept		TM		DHI mol%		Pheomelanin mol%	
	R^2	*p*-value	coefficient	*p*-value	coefficient	*p*-value	coefficient	*p*-value	coefficient	*p*-value
*L**	0.44	8.8×10^{-9}	18.55	3.9×10^{-23}	−0.2215	1.1×10^{-7}	-	-	0.1043	0.042
*a**	0.62	3.5×10^{-13}	8.03	4.1×10^{-5}	−0.1622	8.9×10^{-10}	−0.0522	0.032	0.0756	0.017
*b**	0.58	6.6×10^{-12}	10.16	0.00014	−0.2115	4.1×10^{-9}	−0.0610	0.067	0.0926	0.033

Figure S1

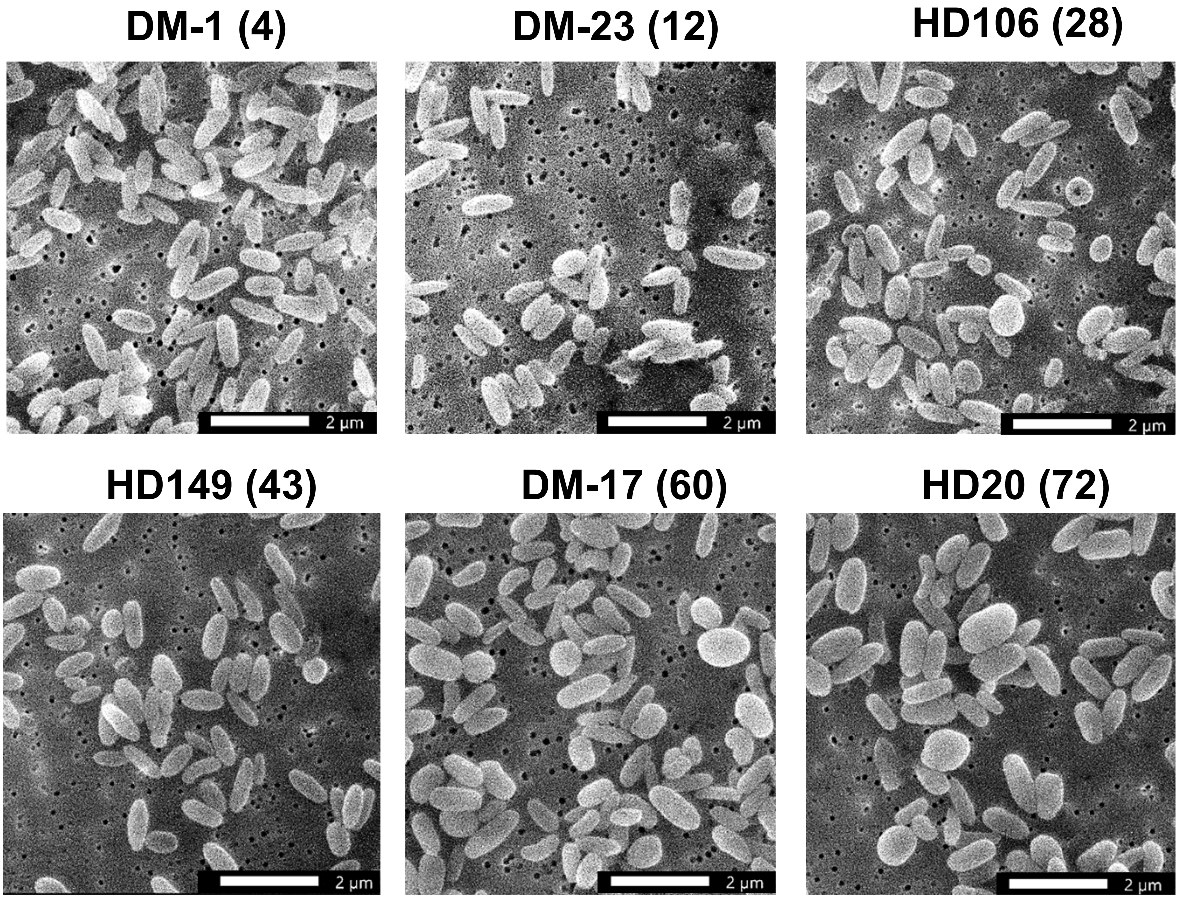

Figure S1 shows representative SEM images of melanosomes isolated from non-chemical-treated Japanese male pigmented hairs. Sample names are shown above the images. Numbers in parentheses are the age of the subjects. The scale bar in each figure means 2 μm and the magnification of all pictures are the same. Isolated melanosomes are ellipsoidal forms on the whole. Small dots with the dimension of 0.1 μm are pores of the membrane.

Figure S2

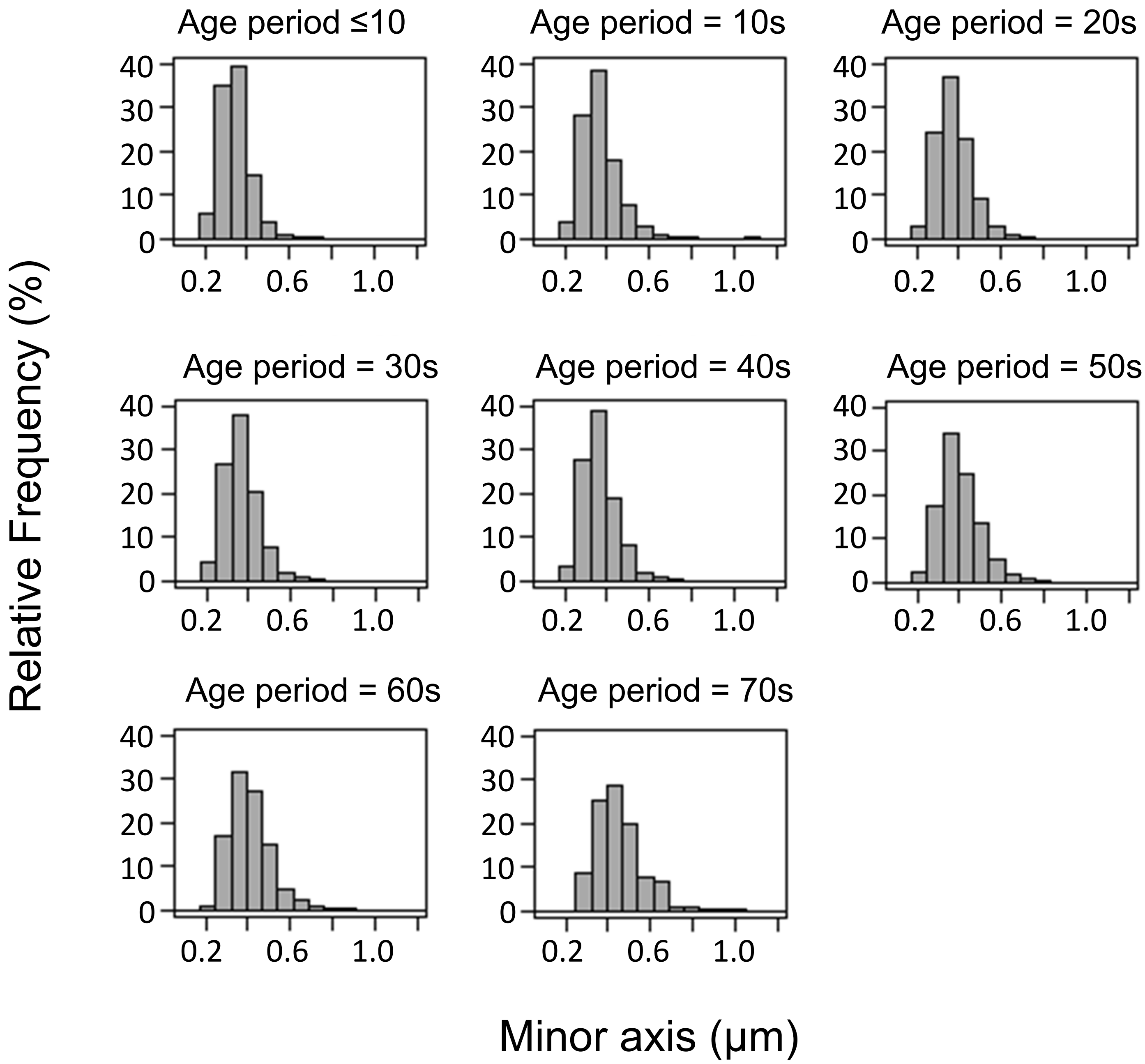

Relative frequency distributions of the melanosomal minor axis for each age period. The age periods are shown above the figures.

Figure S3

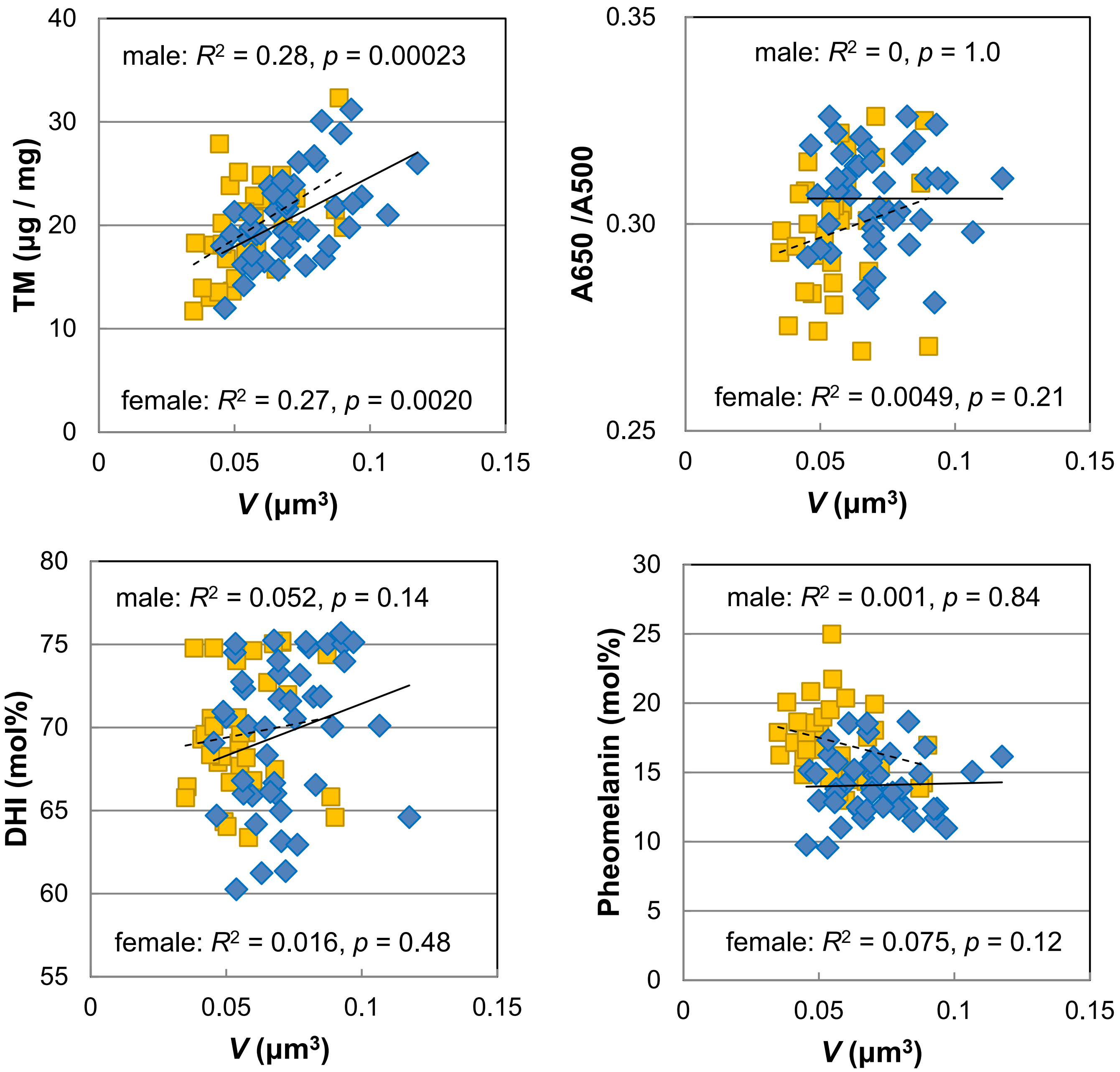

Various values measured by hair melanin analyses vs. mean volume of melanosomes isolated from Japanese male (diamonds) and female (squares) hair samples with an assumption of ellipsoid. (a) Total melanin amount. (b) Absorbance ratio A650/A500. (c) DHI mol%. (d) Pheomelanin mol%. The coefficient of determination and *p*-value are shown in each figure.

Figure S4

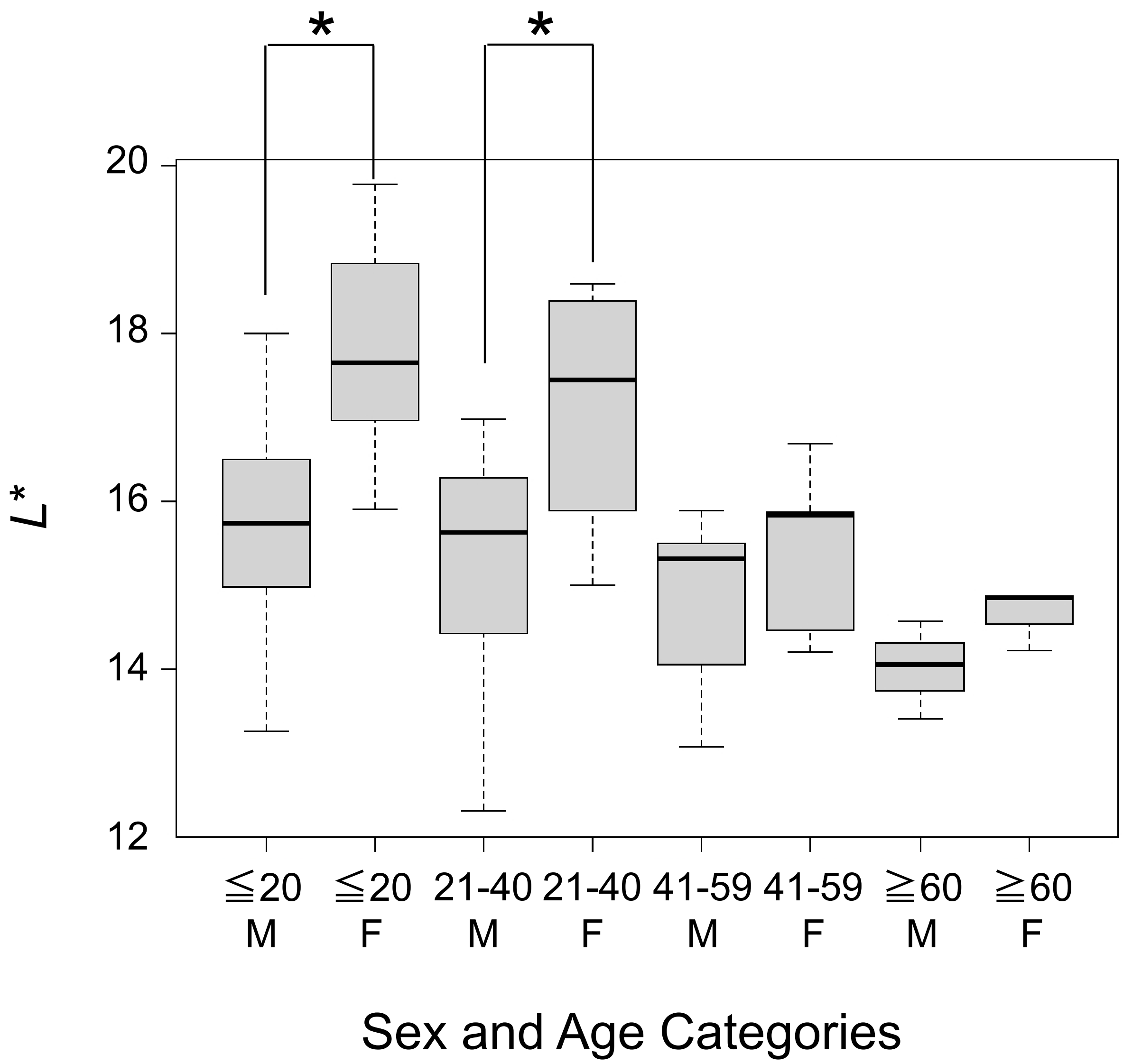

A boxplot of *L** in groups that differ by age period of Japanese male (M) and female (F) pigmented hairs.
* indicates a significant difference at the 5% level.

Figure S5

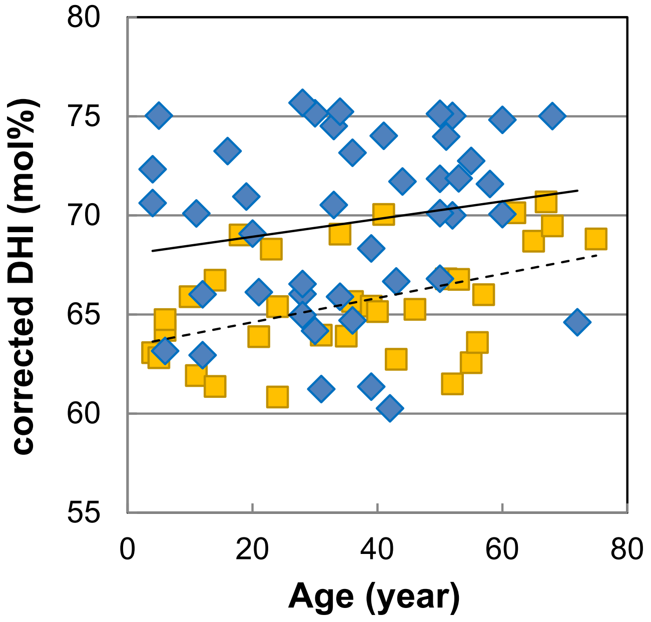

The age dependences of the DHI mol% in Japanese male (diamonds) and female (squares) pigmented hairs. The DHI mol% values for male hairs were measured in this study and those for female hairs were corrected assuming that they would be obtained in the improved HPLC condition (see text).
